# Integration of Immune Cell Populations, mRNA-Seq, and CpG Methylation to Better Predict Humoral Immunity to Influenza Vaccination: Dependence of mRNA-Seq/CpG Methylation on Immune Cell Populations

**DOI:** 10.3389/fimmu.2017.00445

**Published:** 2017-04-21

**Authors:** Michael T. Zimmermann, Richard B. Kennedy, Diane E. Grill, Ann L. Oberg, Krista M. Goergen, Inna G. Ovsyannikova, Iana H. Haralambieva, Gregory A. Poland

**Affiliations:** ^1^Department of Health Science Research, Division of Biomedical Statistics and Informatics, Mayo Clinic, Rochester, MN, USA; ^2^Mayo Clinic Vaccine Research Group, Mayo Clinic, Rochester, MN, USA

**Keywords:** influenza vaccine, data mining, machine learning, cell sorting, methylation, differential expression, immunology

## Abstract

The development of a humoral immune response to influenza vaccines occurs on a multisystems level. Due to the orchestration required for robust immune responses when multiple genes and their regulatory components across multiple cell types are involved, we examined an influenza vaccination cohort using multiple high-throughput technologies. In this study, we sought a more thorough understanding of how immune cell composition and gene expression relate to each other and contribute to interindividual variation in response to influenza vaccination. We first hypothesized that many of the differentially expressed (DE) genes observed after influenza vaccination result from changes in the composition of participants’ peripheral blood mononuclear cells (PBMCs), which were assessed using flow cytometry. We demonstrated that DE genes in our study are correlated with changes in PBMC composition. We gathered DE genes from 128 other publically available PBMC-based vaccine studies and identified that an average of 57% correlated with specific cell subset levels in our study (permutation used to control false discovery), suggesting that the associations we have identified are likely general features of PBMC-based transcriptomics. Second, we hypothesized that more robust models of vaccine response could be generated by accounting for the interplay between PBMC composition, gene expression, and gene regulation. We employed machine learning to generate predictive models of B-cell ELISPOT response outcomes and hemagglutination inhibition (HAI) antibody titers. The top HAI and B-cell ELISPOT model achieved an area under the receiver operating curve (AUC) of 0.64 and 0.79, respectively, with linear model coefficients of determination of 0.08 and 0.28. For the B-cell ELISPOT outcomes, CpG methylation had the greatest predictive ability, highlighting potentially novel regulatory features important for immune response. B-cell ELISOT models using only PBMC composition had lower performance (AUC = 0.67), but highlighted well-known mechanisms. Our analysis demonstrated that each of the three data sets (cell composition, mRNA-Seq, and DNA methylation) may provide distinct information for the prediction of humoral immune response outcomes. We believe that these findings are important for the interpretation of current omics-based studies and set the stage for a more thorough understanding of interindividual immune responses to influenza vaccination.

## Introduction

Goals of vaccine research include improved understanding of vaccine-induced immunity, identification of differences in immune responses to vaccination, and determination of their underlying mechanisms. While vaccination programs to combat seasonal and pandemic influenza strains have been highly effective at decreasing the burden of disease, these gains have not been uniform; specific populations, such as the very young, aged, and immunocompromised, experience the greatest risk for influenza-related complications. It is known that immune responses to vaccination diminish as the age of the vaccinated population increases; however, it has been demonstrated that immune waning is not only strictly correlated with chronologic age but also more closely correlated with molecular measures of immunosenescence (1–4). Thus, leveraging molecular data to enhance our ability to predict response to influenza vaccination is of great interest.

Previous studies of immune response to influenza vaccination have leveraged genetic association and gene expression data to highlight specific pathways and signaling events, which have contributed greatly to our understanding of innate and adaptive immune responses (5–9). A common theme observed throughout these studies is that very few individual genes demonstrate strong effect sizes (5, 10); rather many genes exhibit small effects, similar to what has been observed in genetic association studies for other complex traits (11, 12), including immunity following vaccination (13–16). Thus, to generate robust statistical models of vaccine response, it may be necessary to leverage multiple genetic features whose combined information is greater than each alone.

Many of these previous studies of human immune responses to influenza vaccination have used whole blood (17), or peripheral blood mononuclear cells (PBMCs) (5, 9, 18), to generate transcriptomic data sets to identify the molecular signatures of protective immune responses. However, when high-throughput data are assayed on mixed populations of cells, it is challenging to differentiate changes in population composition from changes in activities within each subpopulation. Methods have been developed for employing immune cell signatures (19, 20) to identify subpopulations of cells within heterogeneous samples, but the interplay between vaccine-induced gene expression changes and interindividual differences in prevaccination PBMC composition is not generally considered. Further, the potentially complementary information among PBMC composition, gene expression, and CpG methylation for constructing predictive models of immune response has not been explored.

To better understand the biologic underpinnings of interindividual immune response to influenza vaccination, we studied a cohort of healthy older individuals (*n* = 159) who received seasonal influenza vaccination. In this study, we first hypothesized that many of the differentially expressed (DE) genes observed upon influenza vaccination are indicative of changes in the composition of PBMCs. Second, we hypothesized that more robust predictive models of vaccine response can be generated by accounting for the interplay between three data types: PBMC composition, gene expression, and gene regulation. To do this, we applied machine learning (ML) techniques and compared our results to other publically available data. We found that the majority of DE genes upon vaccination are associated with changes in PBMC composition. Further, predictive models can be constructed from all three data types, but the highest performing model leveraged all three.

## Materials and Methods

Data used in this study have been made available through http://immunespace.org, under study number SDY67. The following methods are similar or identical to our previously published studies using this cohort (21–26). The primary objective of the original study was to describe and characterize immune response profiles before and after influenza vaccination. In this work, we reanalyze our existing data to integrate across data types by generating novel predictors that leverage markers from each data type.

### Subjects

Subject selection and study recruitment has been previously published (21–23). In brief, the study included 159 healthy individuals, ranging in age from 50 to 74 years old, who were immunized with a single dose of the 2010–2011 seasonal TIV Fluarix (GlaxoSmithKline), containing A/California/7/2009 (H1N1), A/Perth/16/2009 (H3N2), and B/Brisbane/60/2008 viruses (21–23). All subjects reported stable health and provided detailed vaccination histories. Subjects were excluded from the study if they already received the 2010–2011 TIV. Blood samples (90 ml) from each subject were obtained at three separate time points: prevaccination (Day 0), Day 3, and Day 28 (22).

### Flow Cytometry Panels

Our flow cytometry data using fluorescence-activated cell surface marker tags (abbreviated as Flow) consisted of three different panels of cellular/functional and humoral immune markers. The first panel was a measurement of innate immunity (CD11c, CD3, CD86, CD56, CD123, CD20, HLA-DR, CD16, and CD14). The second panel was a measurement of regulatory T-cell phenotypes (CD3, CD4, CD25, CD28, CD38, CD45R0, CD127, CD194, and HLA-DR). The third panel measured B-cell phenotypes (CD3, CD19, CD20, CD24, CD27, CD38, and IgD). Intensity levels were expressed as fractions of the number of total cells that were sorted. Within each panel, data were analyzed for consistency and reproducibility. Cell subset levels were manually reviewed by experienced technicians, and only those with at least 50 cells for each subject in the cohort were carried on to further analysis.

### Immune Assays

The hemagglutination inhibition assay (HAI) has been previously described (21, 24). The standard WHO protocol (27) was used to determine influenza-specific (virus strain A/California/07/2009) antibody titers from each subject’s serum at all three time points. The HAI titer was defined as the highest dilution of serum that inhibits turkey red blood cell (0.5%) hemagglutination. Seroconversion to influenza viral antigens was defined as a fourfold increase in serum antibody titers between Day 0 (before vaccination) and Day 28 (28). The average coefficient of variation for the HAI assay performed in this study was 2.9%.

### B Cell ELISPOT Assay

We have previously described our use of the B cell ELISPOT assay in recent publications (23, 25). In brief, IgG memory-like B cells specific to influenza virus (influenza A/H1N1) were quantified in subjects’ PBMCs using the Mabtech ELIspot^PLUS^ kit for human IgG (Mebtech Inc., Cincinnati, OH, USA), according to the manufacturer’s protocol and as previously described (23, 25). Influenza-specific B cell ELISPOT response was measured in quadruplicate, quantified in spot-forming units per 2 × 10^5^ cells and summarized as subjects’ median. Intraclass correlation coefficients, which assessed the correlation between replicate measurements in this assay, were high (0.88) (23, 25).

### DNA Methylation Assay

DNA samples were extracted and bisulfite modified, and the methylation patterns prevaccination and postvaccination were assessed using the Illumina’s Human 450 Methylation BeadChip, as previously described (26). DNA methylation patterns were measured as percent methylation values (β-value), which were transformed to *M*-values. Our filtering and normalization methods have been previously published and resulted in the interrogation of 101,456 probes across the human genome (26).

### Next-Generation Sequencing

Transcriptome profiling (mRNA-Seq) methods are similar or identical to those we have previously published (24–26). In summary, libraries were prepared from total RNA extracted from PBMCs (all time points), and single-end 50 bp read sequencing was performed on the Illumin HiSeq 2000 (Illumina, San Diego, CA, USA). We used the Illumina Single Read Cluster Generation kit (v2) and 50 Cycle Sequencing Kit (v3). A median of 139.6 million reads per sample were generated. The sequencing reads were aligned to the human genome build 37.1 using TopHat (1.3.3), and Bowtie (0.12.7). HTSeq (0.5.3p3) was used to perform gene counting, and BEDTools (2.7.1) was used to count the reads mapping to individual exons (29–31). The same procedure was applied to purified cell subsets (monocytes, T-cells, and B-cells) from 10 additional subjects (waste blood products from apheresis donors; IRB-approved use). Gene expression features were filtered to those of high interindividual variability (top quartile) and median read count >32 in at least one time point and expressed in log2 units.

### Associations between Flow Cytometry and High-Dimensional Data

We computed the Spearman’s correlation between each high-dimensional feature and Flow features. Because of the large number of correlations and also due to the complex covariance structure within both data sets, correlation coefficients for each Flow feature were computed with 10,000 randomly permuted gene expression features to generate an empirical null distribution. This null distribution was used to filter for significant associations. Correlation coefficient values, observed in up to 1% of random permutations (α = 0.01 level for our empirical null), were filtered. The remaining associations between gene expression profiles and Flow data defined our list of Flow-associated features.

### Public Data Sets

Whole genome DNAse accessibilities, and transcription factor binding site (TFBS) data made available from ENCODE, were downloaded from the UCSC Genome Browser (University of California, Santa Cruz). TFBS narrow-peak calls from all available human ChIP-Seq experiments were downloaded from http://encodeproject.org on 2016-9-23 and filtered to those with maximal confidence (score of 1,000).

We searched GEO (32) data sets for “(PBMC OR “peripheral blood mononuclear”) AND (Vaccine OR virus) AND Taxon:9606.” This query returned 234 data sets that are composed of many different types of comparisons. The utility of these data sets is to investigate if the associations between mRNA and FLOW levels that we have identified are generally observed in PBMC-based studies. We removed data sets with <10 samples and manually reviewed metadata and phenotype tables to identify a control group for differential expression analysis, leaving 186 data sets. We used a semiautomated procedure for identifying DE genes within each study, so that cross-study gene expression normalization was not required. As the dominant trend between conditions in each study could be upregulation or downregulation, we tested a series of fold change thresholds (1/20, 1/10, 1/4, 1/2, 2, and 5) and FDR-adjusted *p*-value thresholds (1 × 10^−1^, 1 × 10^−2^, 1 × 10^−3^, 1 × 10^−4^, and 1 × 10^−5^). For each type of threshold, we identified the most conservative threshold that admitted ≥150 genes. It was necessary to dynamically choose thresholds because different treatments potentially applied within different contexts can produce different magnitudes of fold change. The combined thresholds were then applied to the data set; the resulting DE genes are the intersection of those identified by each threshold. Data sets for which no DE genes were identified according to these criteria were filtered. Finally, the DE genes from across 128 data sets composed of 8,381 samples were compared to the genes significantly correlated with Flow variables in our study. We summarized each study by the fraction of DE genes overlapping with our Flow-associated genes.

We queried ImmuneSpace (33) for human PBMC-based influenza studies that assayed gene expression and ELISPOT outcomes in at least 25 subjects, identifying SDY269 and SDY80. Data were downloaded and processed using the ImmuneSpaceR (34) package. These studies were used for validation of gene expression features identified in our predictive models. No studies beyond our own assayed methylation levels.

### Immune Response Outcomes for Modeling

The primary immune response outcomes used were Day 28 B-cell ELISPOT response expressed on the log2 scale or Day 28 HAI titers expressed on a log2 scale. Continuous levels were modeled, and classification accuracies were determined by splitting the cohort into two groups: “high” and “low.” For B-cell ELISPOT response, we used a median threshold. For HAI, we used a fourfold change from Day 0 levels.

### Clustering Methods

We investigated three clustering methods: *k*-means with *k* = 25, *k*-means with the value of *k* determined by consensus clustering, and WGCNA (35). For each clustering method, we used two procedures for choosing a representative from each cluster—either the cluster’s medoid (i.e., the observation that is closest to the cluster centroid) or the feature with highest correlation with the outcome.

### Generating Predictive Models

To generate predictive models, data were first standardized: ${x^{\prime }}_{i}=(x_{i}-\bar{x})/\text{mad(}x\text{)}$; this is analogous to *Z*-scores, but using medians. Each data set was filtered by a second variance-quartile filter. As a final step prior to any ML, we also removed variables with a Spearman’s correlation coefficient with an outcome less than 0.1; this very low threshold was chosen for noise reduction.

For a given set of input features, we employed 10-fold nested cross-validation (CV) ensemble learner for prediction (36, 37). Ensemble learners are a novel class of ML methods that generate multiple individual models and statistically combine them in a way that minimizes overfitting (38, 39). The ensemble learner used here included individual glm, glmnet, RPART, and random forest models with glmnet used for feature selection within the ensemble. The resulting models were summarized by the number of input features, the number of selected features, and the linear association between the model’s predicted outcomes level and the experimentally measured outcomes. We discretized the fitted and measured outcome levels to evaluate if each model was an accurate classifier for patients having “high” or “low” outcome levels. Classification performance was evaluated using Cohen’s *D, t*-tests, sensitivity, specificity, and area under the receiver operating curve (AUC).

### Software

Analysis was performed using custom scripts in the R programming language (40) version 3.2.0 and leveraging the packages: geosearch (41), geoquery (42), glmnet (43), rpart (44), randomForest (45), Epi (46), and SuperLearner (37). Figures were generated using R and leveraging the ggplot2 (47) and rgl (48) packages.

## Results

### Assay Outcomes

Our study consisted of 159 subjects for which HAI, B-cell ELISPOT, three flow cytometry panels, mRNA-Seq, and CpG methylation data were available at several time points relative to vaccination [details published previously (23–26, 49)]. Two samples were removed due to failed quality control metrics in mRNA-Seq gene expression or CpG methylation.

### Associations between Flow Cytometry Data and Immune Outcomes

Multivariable analysis of our B cell flow cytometry panel data revealed that, in addition to age, the percentage of a combination of cell types including CD8+CD28low T cells (as % of CD8 cells) and the percentages of IgD+CD27− naïve and transitional B cells, CD20− B cells, and CD20-CD27highCD38high plasma cells of total B cells (all measured at Day 3 post-vaccination) were negatively associated with Day 28 HAI response (*R*^2^ = 0.31), as previously described (23). We previously identified specific T and B cell subsets positively associated with Day 28 HAI response (23, 49). While there were no statistically significant changes in plasma cells (CD20−CD27+CD38+) over time, there was a slight percentage increase of B cells at Day 3 versus Day 0 (*p* = 0.006) (23).

### Relationships within and between Flow Data and mRNA Levels

**Figure 1:**
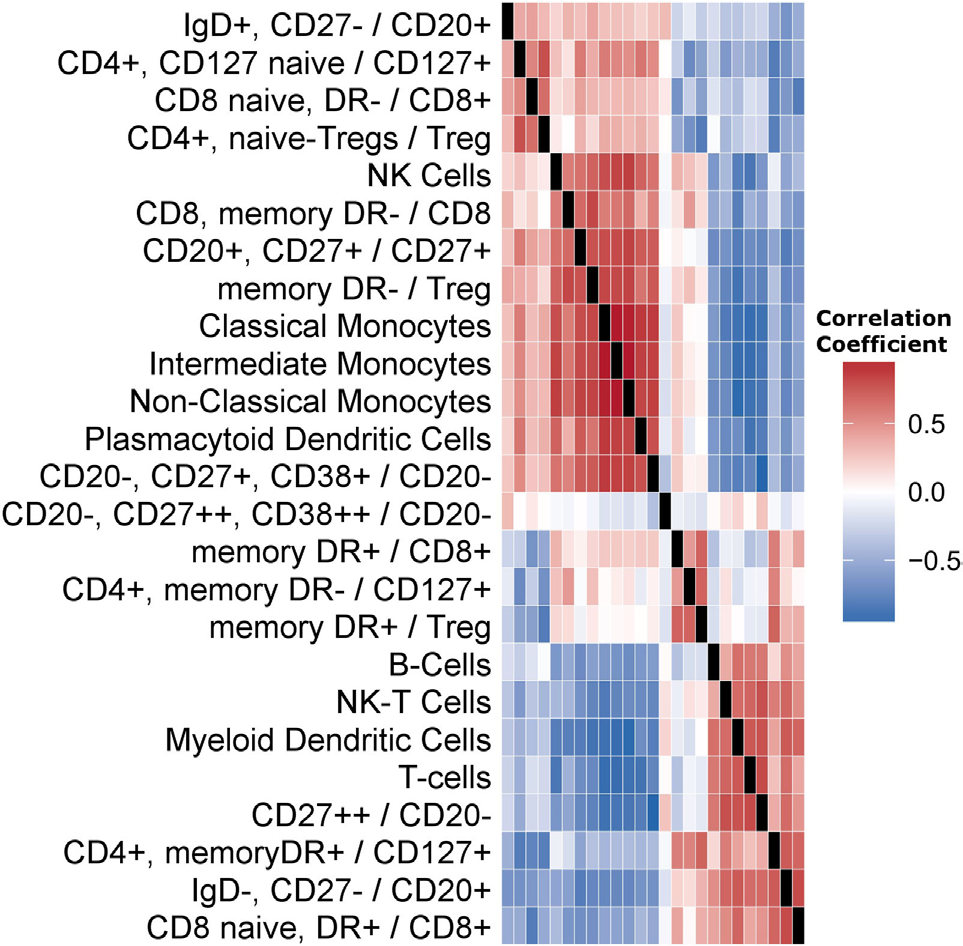
**Correlations among cell subset levels across subjects**. We present a heatmap of Spearman’s correlation coefficients among cell subsets. Each cell in the matrix is the correlation between the corresponding two Flow markers, across subjects. The matrix is symmetric; columns labels omitted for brevity. Row order was determined using hierarchical clustering. Cell subsets are either directly named or labeled by the surface markers used. A forward slash indicates a fraction. For example, the first row indicates the fraction of CD20-positive cells that are IgD positive and CD27 negative.

We first quantified the correlations among cell subset levels (Figure 1). Three groups of cells are visually evident. The first contains NK-cells, plasmacytoid dendritic cells (pDCs), and monocyte subsets. The second is a middle group exhibiting little correlation with other cell subsets, including DR+Tregs and DR+CD8+ memory cells. The third group contained total B-cells, total T-cells, NK-T-cells, and myeloid DCs (mDCs).

**Figure 2:**
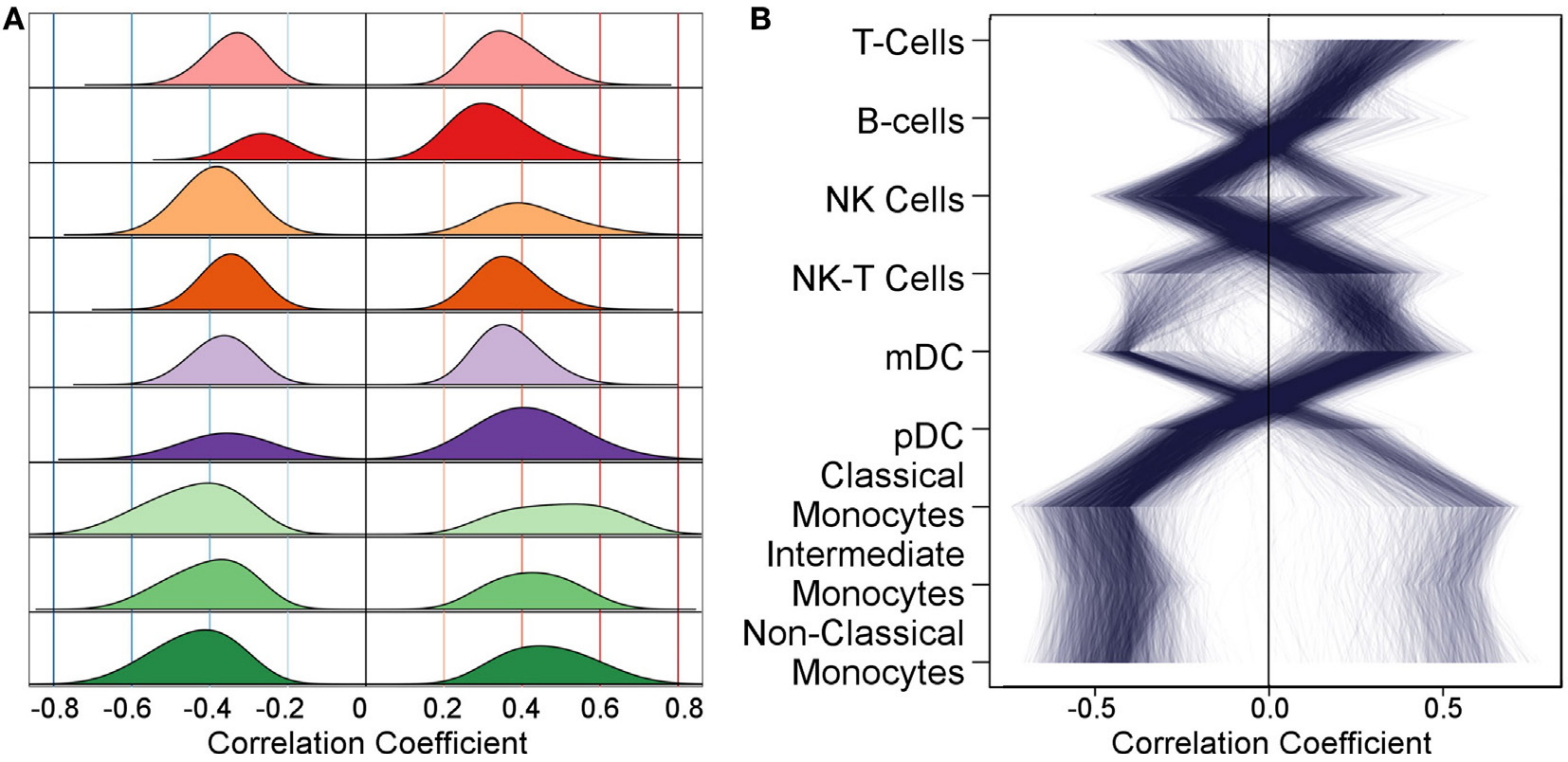
**The distribution of correlations with gene expression differs for each flow cytometry feature**. **(A)** After filtering relationships with low statistical significance using permutation, each cell subset shows positive and negative associations with many genes; each row corresponds to a cell subset. Correlation magnitude is shown along the abscissa and probability density along the ordinate. **(B)** As examples to demonstrate how genes with strong negative correlations with one subset have strong positive correlations with another, we selected genes with correlation ≤−0.4 with any cell subset and plot their associations across all subsets. Each of the selected genes is represented by a line connecting their correlation value with each subset. The same strong trend is observed when selecting genes with a positive correlation coefficient and for smaller magnitudes (not shown).

Next, we correlated gene expression levels with Flow data. Many of the genes that showed differential gene expression exhibited potentially important association(s) with cell subset levels (Figure 2). We used permutation to identify thresholds for likely false-positive associations. The threshold used differed for each subset and were on average 0.24 ± 0.02. The remaining correlations represent 7.8% of all correlations considered. This correlation translates into global gene expression profiles such that variability in immune cell populations over time is correlated with changes in gene expression over time (Figure S1 in Supplementary Material). The analogous trend is not observed for methylation levels (Figure S2 in Supplementary Material). To better understand the relationships between gene expression levels and correlations with Flow subsets, we identified genes with a strong negative correlation (<−0.4) and demonstrate their correlation with all other major innate cell subsets. For example, genes with high correlation with monocyte subsets have the opposite associations with T-cells, NK-T-cells, and mDCs (Figure 2). The number of associated genes differed by cell type (Figure S3 in Supplementary Material). Monocyte populations exhibited the largest number of highly correlated genes, followed by DCs. Further detail is revealed by considering the number of genes whose expression level is correlated with each cell subset level (Figure S4 in Supplementary Material).

**Figure 3:**
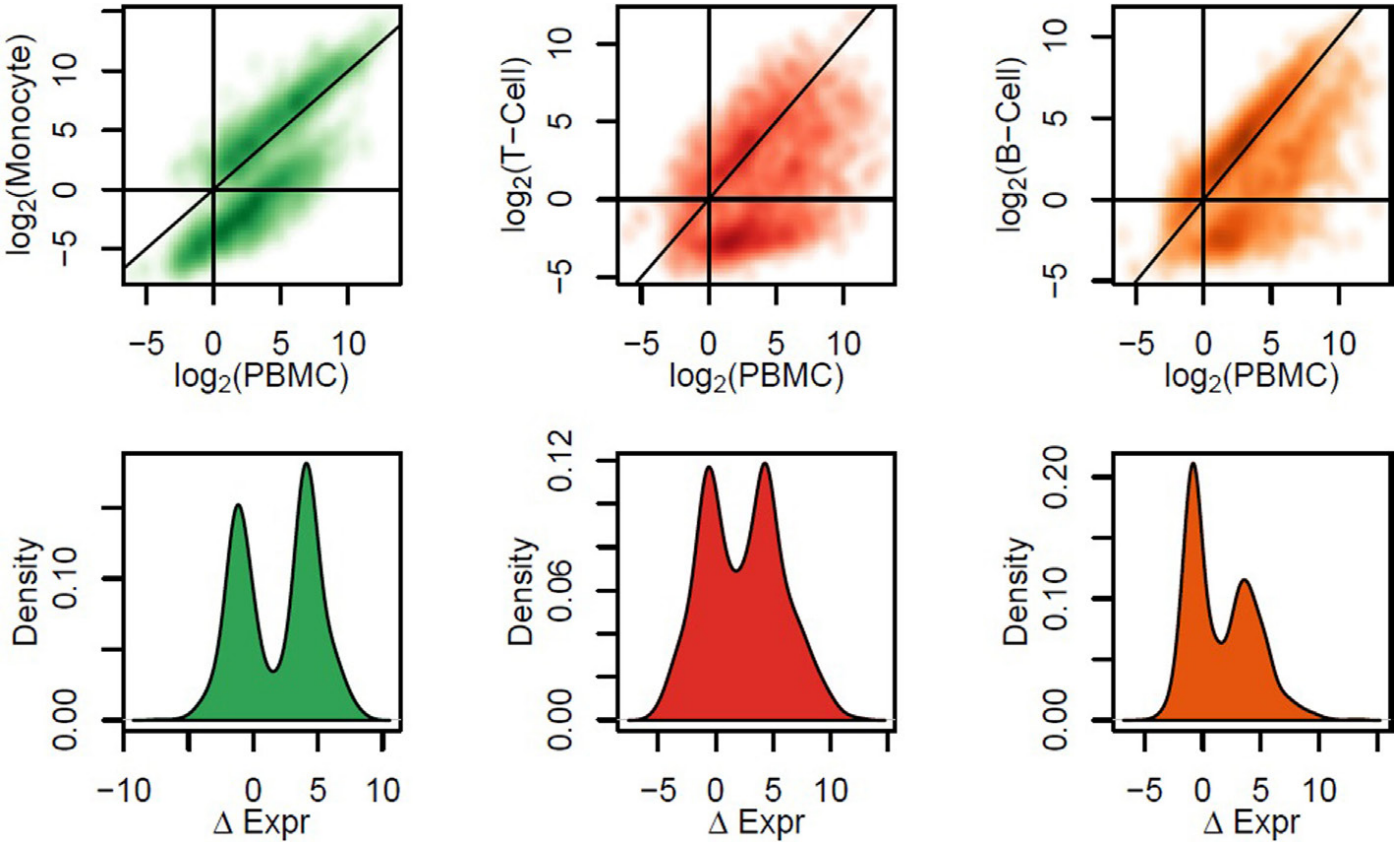
**Comparison between expression levels in human peripheral blood mononuclear cells (PBMCs) and sorted cell subsets**. We performed fluorescence-activated cell sorting for 10 patient samples, and mRNA-Seq was assayed on three sorted cell subsets: monocytes, T-cells, and B-cells. In the first row, we show the relationship between gene expression levels in each cell subset versus PBMCs from the same patient samples, across the most variable quartile of the transcriptome. In the second row, we calculate the difference in expression (ΔExpr) between PBMCs and each sorted cell subset; the probability density of ΔExpr across genes is plotted. These data confirmed the trends observed from data generated on PBMCs—genes correlating with levels of a cell subset according to Flow are expressed to a higher degree in that cell subset than in PBMCs and often than in other cell subsets.

To gain greater resolution on which cell types specifically express each gene, we performed mRNA-Seq on PBMCs and three subsets (fluorescence-activated cell sorting of B cells, T cells, and monocytes) from 10 additional participants. In all three subsets, many genes exhibited significantly different expression levels compared to PBMCs (Figure 3). Many genes exhibited different expression levels in all three subsets. However, the majority of genes with the highest interindividual variability were highly expressed in both B and T cells and lowly in monocytes or *vice versa* (Figure S5 in Supplementary Material). Thus, the identification of which cell subsets drive each gene’s expression is a critical component of understanding the biologic meaning of differential gene expression when assayed in PBMCs.

### Relationships between Flow Data, mRNA Levels, and Immune Response

To assess the degree to which the above associations impact the interpretation of immune response outcomes, we computed the correlation of each Flow-associated gene with B-cell ELISPOT outcomes (Figure S6 in Supplementary Material). T cell and pDC subset genes have the highest proportion of expression-associating genes with significant associations (*p* < 1 × 10^−2^) with immune response levels. About half of the monocyte- and NK-T-associated genes are also associated with B-cell ELISPOT outcome levels, while only few B cell- and NK cell-associated genes are associated with B-cell ELISPOT outcome levels.

**Figure 4:**
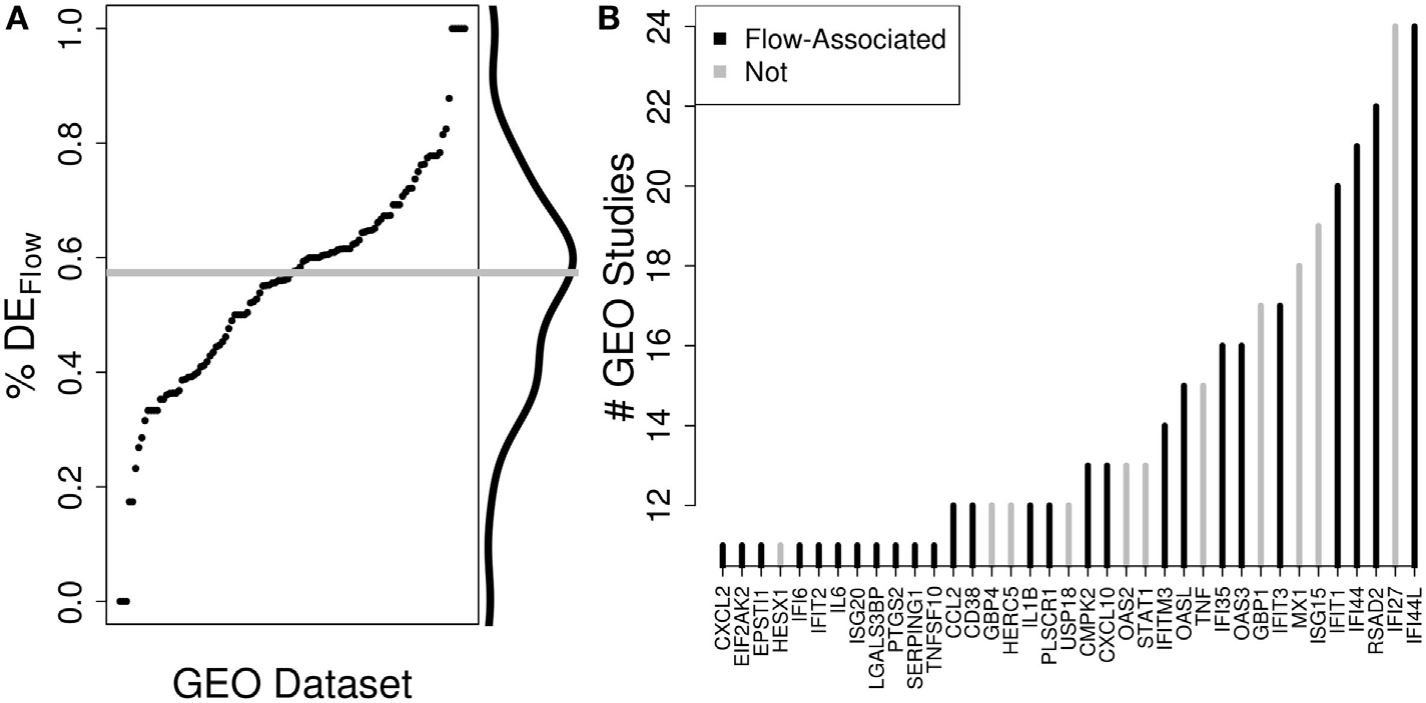
**The potential impact of accounting for immune cell composition on the interpretation of peripheral blood mononuclear cell (PBMC)-derived gene expression profiles**. We quantified the fraction of differentially expressed (DE) genes from publically available studies for which the same gene was strongly correlated with an immune cell subset in our study. **(A)** Across 128 publically available human vaccine-related data sets where data were produced from PBMCs, we identified DE genes and determined the percent of those DE genes that are significantly associated with Flow-derived cell subset levels in our data set (%DE_Flow_). On average, 57% of the DE genes from external PBMC-derived samples were associated with a change in cell subset level in our study. **(B)** Recurrence analysis of DE genes across these studies highlights that underlying changes in PBMC composition could be driving many of the most important transcriptomic changes across these studies.

To validate the potential impact of these relationships on the interpretation of PBMC-derived profiles, we examined DE genes from publically available data sets of vaccine or virus response assayed in PBMCs and identified 57% overlap, on average, with the genes exhibiting strong correlations in our study (Figure 4). Thus, it is likely that the association between Flow variables and gene expression is a general property of PBMCs. These associations should be considered for the interpretation of high-dimensional data assayed on PBMC samples.

### Predictive Models of Immune Response

Given the interdependencies evident within and between our three data sets, the utility of combining them into a more powerful predictive model of immune response was investigated. We will refer to variables from each data set generically as “features” for model development. Different combinations of data sets were submitted to an ensemble learner, a ML algorithm that combines multiple marginal predictions so as to maximize performance while not significantly affecting the rate of false discovery, compared to other ML algorithms (36, 39).

#### Models Built from Single-Data Types

Individual data sets demonstrated different abilities to produce predictive models for B-cell ELISPOT response. We have summarized prediction results across all models in Table 1. Flow variables alone achieved high sensitivity, but low specificity, resulting in a modest AUC of 0.67. The three features retained by the Flow-only model, selected from all available Flow features plus age and biologic sex, were the levels of B-cells, DR− memory Tregs, and the fraction of CD20+ cells that are CD27+. Thus, ML identified these specific cell types as important for memory B-cell immune response. Gene expression alone exhibited less robust prediction by multiple measures; the model had an AUC of 0.51 and contained six genes: *BHLHE41, DES, NXPH4, NOMO3, PKIB*, and *TKTL1*. Changes in these genes’ expression levels are associated with, respectively, the levels of the following cell subsets: mDCs, monocytes, B-cells, monocytes and DR− memory Tregs, monocytes, and NK-cells. Differential expression alone also yielded less robust prediction (AUC = 0.55). Two genes were selected by this model and also showed correlations with Flow levels: *HSD17B14* correlated with classical monocytes and pCDs, while *MACROD2* correlated with mDCs and T cells. Methylation alone achieved an AUC of 0.78 and demonstrated greater separation of high and low responders than other per-data type models. Detailed performance metrics for all models were examined, and examples are available in Figure S7 in Supplementary Material. Thus, per-data type models indicate that PBMC composition and CpG methylation may provide complementary information for prediction of immune response outcomes.

**Table 1:** **Performance of predictive models of B-cell ELISPOT using combinations of data types**.

Input data			Feature selection		Continuous prediction		Discrete prediction^a^				
Flow	mRNA	CpG	*N*	*M*	LM *r*^2^	LM *p*-value	*D*^b^	*t*-test	sens	spec	AUC
*F*_0_			27	3	0.07	1.05 × 10^–3^	0.67	1.59 × 10^−4^	0.78	0.53	0.67
	*E*_0_		151	6	0.00	7.73 × 10^−1^	0.01	7.60 × 10^−1^	0.75	0.35	0.51
	*F*_0_^C^		31	1	0.00	4.81 × 10^−1^	0.02	3.68 × 10^−1^	0.30	0.79	0.52
	*E*_28-0_		63	2	0.04	1.83 × 10^−2^	0.11	2.61 × 10^−1^	0.44	0.69	0.55
	*E*_28-0_ ∩ *F*_0_^C^		10	3	0.00	5.38 × 10^−1^	0.00	8.40 × 10^−2^	0.90	0.22	0.55
		*M*_0_	72	29	0.23	1.81 × 10^−10^	1.13	1.21 × 10^−10^	0.76	0.73	0.78
*F*_0_	*E*_0_		178	8	0.03	2.74 × 10^−2^	0.52	4.91 × 10^−3^	0.58	0.68	0.63
*F*_0_	*F*_0_^C^		58	3	0.04	1.14 × 10^−2^	0.44	3.67 × 10^−3^	0.65	0.62	0.63
*F*_0_	*E*_28-0_		90	4	0.03	4.22 × 10^−2^	0.49	9.25 × 10^−3^	0.58	0.67	0.62
*F*_0_	*E*_28-0_ ∩ *F*_0_^C^		37	3	0.06	1.97 × 10^−3^	0.68	1.38 × 10^−4^	0.65	0.65	0.68
*F*_0_		*M*_0_	99	31	0.22	5.77 × 10^−10^	1.04	4.29 × 10^−11^	0.81	0.67	0.79
*F*_0_	*E*_0_	*M*_0_	250	35	0.12	1.22 × 10^−5^	0.84	3.03 × 10^−5^	0.63	0.69	0.69
*F*_0_	*F*_0_^C^	*M*_0_	130	27	0.17	5.42 × 10^−8^	1.06	4.29 × 10^−9^	0.82	0.63	0.76
*F*_0_	*E*_28-0_	*M*_0_	162	32	0.28	1.63 × 10^−12^	1.08	2.26 × 10^−11^	0.70	0.76	0.79
*F*_0_	*E*_28-0_ ∩ *F*_0_^C^	*M*_0_	109	29	0.19	8.96 × 10^−9^	1.01	4.04 × 10^−9^	0.71	0.74	0.76
**Best representative^c^**											
	*E*_0_, *k* = 25	*M*_0_, *k* = 25	50	32	0.22	7.42 × 10^−10^	0.84	2.59 × 10^−7^	0.62	0.76	0.73
*F*_0_	*E*_0_, *k* = 25	*M*_0_, *k* = 25	77	23	0.18	3.05 × 10^−8^	0.88	4.47 × 10^−7^	0.72	0.68	0.72
	*E*_0_, *k* = 6	*M*_0_, *k* = 8	14	10	0.15	8.49 × 10^−7^	0.75	9.68 × 10^−7^	0.72	0.65	0.72
	*E*_0_, WGCNA = 15	*M*_0_, *k* = 8	23	13	0.13	3.13 × 10^−6^	0.80	1.27 × 10^−6^	0.68	0.65	0.71
*F*_0_	*E*_0_, *k* = 6	*M*_0_, *k* = 8	41	10	0.13	3.27 × 10^−6^	0.83	9.51 × 10^−7^	0.62	0.74	0.72
**Medoid representative**											
	*E*_0_, *k* = 25	*M*_0_, *k* = 25	50	7	0.01	2.36 × 10^−1^	−0.19	3.06 × 10^−1^	0.61	0.56	0.56
*F*_0_	*E*_0_, *k* = 25	*M*_0_, *k* = 25	77	3	0.06	1.97 × 10^−3^	0.43	2.80 × 10^−3^	0.37	0.86	0.64
	*E*_0_, *k* = 6	*M*_0_, *k* = 8	14	2	0.03	2.24 × 10^−2^	0.30	2.77 × 10^−2^	0.59	0.62	0.60
	*E*_0_, WGCNA = 15	*M*_0_, *k* = 8	23	3	0.02	9.50 × 10^−2^	0.29	4.88 × 10^−2^	0.85	0.32	0.59
*F*_0_	*E*_0_, *k* = 6	*M*_0_, *k* = 8	41	3	0.05	3.28 × 10^−3^	0.60	2.88 × 10^−4^	0.80	0.50	0.67

*AUC, area under the receiver operating curve; *F*_0_, day 0 cell subset levels (flow) plus participant age and biologic sex; *E*_0_, day 0 gene expression; *F*_0_^C^, day 0 gene expression of genes not correlated with any flow data; *M*_0_, day 0 methylation; *N*, the number of features input to the ensemble learner; *M*, the number of features retained in the final model; LM, linear model fit between observed and predicted outcome levels*.

*^a^For evaluative purposes, outcome data were discretized by above or below the median*.

*^b^Cohen’s D statistic measuring the standardized difference in means*.

*^c^Data were clustered into k clusters before model construction, and one representative from each was used*.

#### Models Built from Multiple Data Types

First, we combined the aforementioned mRNA and Flow features and generated a new model, which included naïve CD4+Treg and IgD+CD20+CD27+ B-cell levels and expression of additional genes, including *BHLHE41, NOMO3, PKIB*, and *TKTL1*. With these additional features, moderate gains in performance were observed—particularly an increased specificity (Table 1). Combining Flow and methylation data resulted in 27 of the original 29CpGs selected, with the three cell subsets from the Flow-only model. Importantly, nearly all models generated that utilized Flow features retained the three cell subset variables identified in the Flow-only model. The only two exceptions were models of Day 0 gene expression with or without Day 0 methylation that did not include B-cell levels; however, genes were included in these models whose expression levels were correlated with either B-cell or T-cell levels. Thus, the complementary information between Flow and CpG methylation was further supported.

Combining features from all three data types achieved the highest specificity and AUC. The model was composed of nearly identical features to the Flow and methylation model, with included expression levels of five genes: *BTNL9, HSD17B14, MACROD2, OXTR*, and *UGT8*. As previously stated for the expression-only model, *HSD17B14* and *MACROD2* are associated with T-cells and mDCs and B-cells and mDC, respectively. *BTNL9* and *UGT8* are correlated with B-cell levels and *OXTR* was not significantly associated with Flow levels. Therefore, Flow and methylation provided the predominant signal in our model, with expression of genes typically correlating with cell subset levels providing modest improvements.

Finally, and because of the extensive correlation structure present within each data set (Figure 1; Figure S8 in Supplementary Material), we tested the effect of using different clustering techniques. First, we used *k*-means clustering and chose a representative from each cluster to be given to the ML procedure. Representatives were chosen in one of two ways: the “central” feature (medoid) from each cluster versus the feature with strongest association with outcome. Second, we used consensus clustering approaches to optimize the number of clusters chosen. The best performing model across all methods considered was from clustering prior to feature selection; however, models generated from cluster representatives shared a significant number of features with the models generated without clustering. For example, *BHLHE41, NOMO3, NXPH4, PKIB*, and *TKTL1* gene expression levels were frequently included in predictive models of B-cell ELISPOT response. These genes are all strongly correlated with the levels of cell subsets, emphasizing the interrelated nature of high-dimensional data and sample composition.

The same analysis approach was applied to the prediction of HAI response (Table S1 in Supplementary Material), but was less successful at identifying high and low responders regardless of which data types were used. The most predictive model was Day 0 gene expression of genes not correlated with any Flow data (Cohen’s *D* = 0.49, AUC = 0.64).

### Comparison to Other Data Sets

While no comparable data sets of methylation levels and influenza vaccination were found for validation, the ImmPort database contained several data sets for gene expression before and after influenza vaccination. Prevaccination gene expression from three cohorts [SDY269-TIV (9), *n* = 28; SDY269-LAIV, *n* = 28; and SDY80 (5), *n* = 51] was used to investigate the immune response outcome associations for the top 10 genes from predictive modeling in our study (Figure S9 in Supplementary Material). These comparison data were acquired on microarray, and no probesets interrogated *NOMO3*. Further, these studies are smaller than ours and use different technologies, reagents (e.g., antibodies and vaccines), and procedures. Subjects in SDY80 received both seasonal influenza and H1N1 vaccines. Subjects in SDY269 received either LAIV (FluMist) vaccine, likely to activate the immune system differently from TIV vaccines, or a TIV vaccine, but were recruited over three seasons and assayed using plasmablast ELISPOT—an immune response outcome expected to be correlated with our B-cell ELISPOT outcome, but the strength of correlation is unknown. Thus, these previous studies are not fully comparable to ours, and the SDY269-TIV cohort is likely to most comparable. Evaluating the nine genes identified in our analyses demonstrated the greatest reproducibility in SDY269-TIV, but variable reproducibility in SDY269-LAIV and SDY80. However, statistically significant predictive models could be generated for all three data sets using only the nine genes (Figure S9 in Supplementary Material), indicating the potential for these genes to provide information about immune response outcomes.

### Annotation of Model Features

Many of the same genes and CpGs were selected by our ML procedure for inclusion in multiple predictive models. We list the occurrence of features across our predictive models in Table S2 in Supplementary Material. Flow levels and CpG sites lie at the top of this list. To facilitate the interpretation of the potential biologic mechanisms that these CpG sites may be indicative of, we annotated them for their potential regulatory roles by their relationship to genes, DNA accessibility, and integrated TFBS measurements from ENCODE ChIP-Seq data (Table 2). About half of them are *cis*-acting to a gene, lying either within the gene’s promoter or within the gene body. Nearly all of them overlap known ChIP-Seq-identified TFBSs and are accessible *via* DNAse digestion. Many of the TFs identified at each site are known chromatin remodeling enzymes (e.g., CTCF, MYC, MAX, SIN3A, EP300). Differential methylation at these sites could influence either immune cell composition or activity through differential binding of these chromatin remodelers at the same sites, potentially influencing the regulation of multiple genes. Thus, mechanistic hypotheses for the role of each CpG site in specific TF binding events, and therefore, gene regulation are apparent.

**Table 2:** **Annotation of CpGs recurrently used in classification of B-cell ELISPOT outcomes**.

Illumina ID	Context	NC^a^	Promoter	Body	DNAse^b^	#TF^c^	Transcription factor binding site (TFBS)^d^
cg06739303	S_Shore	6	LOC441666		x	46	ELF1, FOS, GABPA
cg17959722	Island	6	PNPLA7		x	28	E2F1, POLR2A, SIN3A
cg19566405		6	SLFN12		x	16	ZNF263, FOS, JUND
cg00310523		6	RASSF9		x	11	CEBPB, TBP, TCF7L2
cg18963800	N_Shore	6	HSD17B7P2		x	7	CEBPB, POLR2A
cg21384492	Island	6	SNED1			3	E2F1, POLR2A, SIN3A
cg15878909		5	FAM90A1		x	9	MAX, POLR2A, RAD51
cg00785941	Island	5	OR2L13			31	CTCF, ZNF263, ELF1
cg15633073	Island	11		ZNF536	x	0	
cg20550154		6		NID2	x	8	EP300, NFE2, ZNF384
cg00367615	Island	6		MEDAG	x	1	EZH2
cg04681845		6		FMNL2	x	1	MYC
cg18396987	S_Shore	6		SYCP1		1	EZH2
cg11430096	S_Shore	6		CDK19		0	
cg18498565		3		PFKP	x	8	CEBPB, FOS, EP300
cg08065408	N_Shore	11			x	5	NFYB, RFX5, ZBTB40
cg14521995	S_Shore	11			x	0	
cg03532030	S_Shore	10			x	27	MAX, POLR2A, SPI1
cg02599498		6			x	69	EP300, JUND, MYC
cg15203566	Island	6			x	7	RAD21, TBP, EZH2
cg17292337		6			x	6	E2F6, L3MBTL2, EZH2
cg11757417		6			x	0	
cg06470855	Island	6			x	0	
cg19510820		6				1	MAFK
cg16005559	S_Shore	5			x	0	
cg18307968		5				5	CTCF, MAFK, MAFF
cg06134410	Island	3			x	2	E2F6, UBTF
cg03121508		3				1	EZH2

*^a^The number of classifiers that selected the CpG site*.

*^b^DNAse sensitivity made available through UCSC; an “x” indicates an accessible site*.

*^c^Number of unique TFBSs overlapping the loci from ENCODE*.

*^d^Highest scoring TFBSs overlapping the loci; up to three are shown for brevity*.

## Discussion

Applying systems biology approaches, high-dimensional data sets, and advanced analytical tools is critical to furthering our understanding of human immune responses to infection and/or vaccination (50). Transcriptomic analyses are being used to examine human immunology; however, the integration of transcriptomic data with additional data types may improve our ability to assess immune responses and potentially generate improved predictive models. In this study, we analyzed the extent to which gene expression and methylation level changes are influenced by PBMC composition (assessed by flow cytometry) and applied ML to combine these three data types in different ways to construct predictive models of immune response to seasonal influenza vaccination.

Many systems biology studies of human immune responses have used PBMC samples to generate high-throughput data sets to identify molecular signatures of robust immune response. A key advantage to this approach is that blood is easily obtained from individuals and PBMCs can be isolated without intensive purification procedures that potentially alter cellular gene expression patterns. In addition, whole-cell samples likely better reflect holistically the *in vivo* environment in which immunity is generated. A disadvantage is that PBMCs are a diverse mixture of cell types, each with a potentially unique gene expression response to a given stimulus. A common theme observed throughout high-throughput studies utilizing PBMCs has been that few individual genes contribute consistently to immune responses and those that do have relatively small effect sizes. This is likely not only due to the fact that immune responses are complex, multigenic processes but also due to the presence of multiple, cell subset-specific responses to the vaccine that are superimposed.

While it is intuitive that changes in PBMC composition would lead to changes in PBMC-derived gene expression, it is not taken into account by the majority of studies. An immune response is the result of a complex interaction of numerous components working in a coordinated fashion to elicit immune response. Thus, by its nature, interdependencies within and between data sets should be expected. In this study, we have identified many associations between the cellular composition and resulting transcriptomic data from PBMC samples. Interestingly, less prevalent cell populations, such as mDCs, tend to have many gene expression changes associated with their population-level changes, compared to more prevalent cell types. Thus, the relative expression levels of each gene and between cell types may be an additional factor for consideration. The associations found in our data set with specific genes’ expression levels are recapitulated within multiple publically available data sets assayed on PBMCs, which makes accounting for them potentially important for the interpretation of results, as well as the interpretation of predictive models generated from the same data.

In principle, studies should assay gene expression within multiple subsets to attain the highest resolution, but this is often not feasible. Flow analysis is a strong companion assay to help interpret differential gene expression. We recommend checking how expression profiles associate with Flow levels and if the simplest interpretation of the data is predominantly through changes in PBMC composition, or through gene expression changes within a specific cell subset. When analysis of specific cell subsets is not an option, computational deconvolution may be helpful (19, 20). If possible, we believe that performing assays in the most applicable cell subset will yield more clearly interpretable results than in mixed populations of cells. However, when no clear candidate cell subset exists, and/or when searching for biomarkers, or due to cost considerations, PBMC-based studies may be appropriate.

Genes that had expression levels associated with multiple cell subsets recapitulated expected relationships, such as the progression between classical, intermediate, and non-classical monocytes, but also revealed further relationships (Figure S4 in Supplementary Material). One example is the high overlap in gene expression between T and NK cells, but the lack of overlap between both of these subsets and NK-T cells. Due to NK-T cells sharing properties with both NK and T cells (IFN-g secretion, perforin/granzyme secretion, expression of death-inducing receptors/ligands: TNF-TNFRs, TRAIL-TRAILRs, and FAS-FASL), one may expect them have an intermediate gene expression profile. A recent paper demonstrated that murine NKT1 cells shared transcriptomic similarities with NK and Th1 cells, but that NKT2 and NKT17 cells did not (51). This example highlights the incredible cellular complexity of blood leukocyte populations. Thus, a more distinct gene expression pattern of NK-T cells is indicated by our analysis and may support further investigation of the detailed differences among subpopulations within defined cell subsets (T cells, B cells, NK cells, etc.) (52).

We generated predictive models using each data set (Flow levels, mRNA expression, and CpG methylation) and combinations of data sets, comparing the predictive performance of each, as well as the types of features used by each model. Age and biologic sex were included as candidate features in all models that leveraged flow cytometry data; however, the ML model did not select age or sex in cross-validated models, likely due to their known correlations with genetic features (4, 53–55). Flow levels were critical in all expression-based models, either through their direct inclusion or through the interpretation of gene expression features. Three cell subsets were repeatedly included in models: B-cells, memory Tregs, and CD20+CD27+ cells (plasmablasts). These three subsets efficiently capture aspects of a subject’s adaptive capacity, prior exposure, and activated memory-based response. All genes (besides *OXTR*) included in predictive models were correlated with changes in Flow levels—B-cell levels in particular. Methylation was the most predictive of the single-data type models. Nearly all predictive CpG sites are within open chromatin states and overlap known TFBSs (Table 2). Thus, they have a capacity to regulate gene expression, but which genes are primarily affected may require further study. We believe a component of the predictive nature of methylation levels could be through their influence on differentiation rates of certain cell subsets.

Our DNA methylation data highlighted CpG sites within several genes (Table 2) that are associated with variations in memory B cell ELISPOT response to influenza vaccination. Interestingly, PNPLAA7, MEDAG, CDK19, and PFKP are all involved in metabolic activity (mostly lipid and glucose metabolism). Examination of transcriptomic data in this same cohort identified cholesterol and lipid metabolism-related genesets associated with memory B cell ELISPOT responses after influenza vaccination (25). Other groups have also reported on metabolic changes in B cells associated with activation and antibody production (56, 57). The DNA methylation findings reported here support those previous findings and provide a potential mechanism for the observed changes in gene expression. B cell metabolism during activation and acquisition of effector function has not been well studied. Our results suggest that the metabolic activity of B cells may contribute to differential vaccine responses and that further investigation into this area may improve our understanding of how humoral immune responses to vaccines are regulated.

Our studies examining the response to seasonal influenza vaccination were carefully designed, and data acquisition was performed by experienced core labs. Previous studies of vaccine response that used high-throughput technologies, such as gene expression and CpG methylation, were performed in smaller numbers of subjects than our current study. Thus, while we are challenged by an overall cohort sample size (*n* = 159), our data are of high quality and have minimal systematic and technical noise, as our previous work has addressed (25, 58). We performed aggressive data filtering prior to generation of any ML models; thus, models using high-throughput data did not start from many thousands of features, but from hundreds of features that were the most variable across samples.

The standard approach for validation is to generate a model within one cohort and then, after it is finalized, test it in an externally derived cohort consisting of similar samples. Where the external cohort comes from is an important consideration. One option is to collect and process samples, but leave a group of them out of the analysis. Thus, they are known to be comparably processed and can be controlled for clinical similarity. CV seeks to perform this type of left-out validation in a more statistically powerful way wherein models are tested on sections of the data set that were not used in training. It is critical that data used in training and testing are never shared during each loop of CV. Few of our cohort, data type, and vaccine-type characteristics were strong matches with previously published studies, making orthogonal retrospective validation challenging; therefore, we employed a ML approach to identify the most robust associations within our data set.

Our study emphasizes some of the challenges of applying high-dimensional technologies to the study of immune response outcomes to vaccines. While our cohort is among the largest used in vaccinomics studies, limitations of statistical power and data interpretation due to correlations between data types remain. In this study, we have integrated data types to assess these challenges and applied ML methodologies to address them. The genes identified in our study are known to impact immune response outcomes, but here we have demonstrated that they may also be markers of differences in immune cell composition. This indicates that gene expression studies performed on mixed cell populations must take into account the interindividual differences in cell subset makeup to accurately interpret their results. These findings also suggest that studies on purified cell populations may result in stronger, more readily detectable, changes in gene expression or regulation. Better predictive models could be generated using CpG methylation sites, providing compelling candidates for future studies to determine their potential in directly regulating immune response mechanisms, in regulating immune cell composition through signals of cellular differentiation, or as markers of a subject’s potential to respond. Importantly, our study utilized established immune response outcomes to vaccination. We have shown that genomic features can be used to generate predictive models of those outcomes. Our cohort size allowed us to use nested CV to optimize the potential reproducibility of the model. Taken together, we believe that these data highlight important considerations for data integration and the interpretation of high-dimensional data for immune response outcomes.

## Conclusion

We have found that (1) overall variability of participants’ PBMC composition is correlated with overall variability in gene expression, (2) many of the individual genes with statistically significant gene expression changes are associated with changes in specific cell subsets, and (3) PBMC composition is a strong predictor of humoral immune response. While the importance of immune cell composition is known, high-dimensional data can provide further information to improve prediction of immune response to vaccination, and these features may improve our understanding of the underlying mechanisms of interindividual response variations. Using the largest available data set of high-quality humoral immune outcomes to influenza vaccination, paired with genomic and epigenomic data, we have identified that predictive models leveraged features of immune cell composition and CpG methylation. Therefore, the high-dimensional features identified by our predictive modeling approach may indicate regulatory mechanisms that are active in modulating immune responses including alteration in PBMC composition. We believe that these findings, which emphasize the strong interplay between sample cell composition and high-dimensional data, are important for the interpretation of current omics-based studies and, when applicable, should be accounted for within ongoing studies of immune responses.

## Ethics Statement

All subjects provided written informed consent. This study was approved by the Mayo Clinic Institutional Review Board.

## Author Contributions

MZ planned the study, performed analyses, and wrote the paper. AO, DG, and RK planned the study and wrote the paper. KG performed analyses. IH, IO, and GP wrote the paper. All the authors contributed critical review of the manuscript.

## Conflict of Interest Statement

GP is the chair of a Safety Evaluation Committee for novel investigational vaccine trials being conducted by Merck Research Laboratories. GP offers consultative advice on vaccine development to Merck & Co. Inc., Avianax, Dynavax, Novartis Vaccines and Therapeutics, Emergent Biosolutions, Adjuvance, Seqirus, and Protein Sciences. GP and IO hold three patents related to vaccinia and measles peptide research. GP and RK hold a patent related to vaccinia-derived epitope research. RK has received grant funding from Merck Laboratories to study waning immunity following mumps vaccination. These activities have been reviewed by the Mayo Clinic Conflict of Interest Review Board and are conducted in compliance with Mayo Clinic Conflict of Interest policies. This research has been reviewed by the Mayo Clinic Conflict of Interest Review Board and was conducted in compliance with Mayo Clinic Conflict of Interest policies. No other coauthors have competing interests to declare.
