# Supplementary material for: Integration of Immune Cell Populations, mRNA-Seq, and CpG Methylation to Better Predict Humoral Immunity to Influenza Vaccination: Dependence of mRNA-Seq/CpG Methylation on Immune Cell Populations
Source: Front Immunol. 2017 Apr 21;8:445. doi: 10.3389/fimmu.2017.00445 (PMC5399034; doi:10.3389/fimmu.2017.00445)
Supplement: Supplementary file 1 [file data_sheet_1.docx]

Supplementary Information to:

**Integration of Immune Cell Populations, mRNA-Seq, and CpG Methylation to Better Predict Humoral Immunity to Influenza Vaccination: Dependence of mRNA-Seq/CpG Methylation on Immune Cell Populations**

M.T. Zimmermann^1,2^, R.B. Kennedy^2^, D.E. Grill^1,2^, A.L. Oberg^1,2^, K.M. Goergen^1^, I.G. Ovsyannikova^2^, I.H. Haralambieva^2^, G.A. Poland^2,*^

**Affiliations:**

^1^Department of Health Science Research, Division of Biomedical Statistics and Informatics, Mayo Clinic, Rochester, MN, 55905, USA.

^2^Mayo Clinic Vaccine Research Group, Mayo Clinic, Rochester, MN, 55905, USA.

^*^Corresponding author

**Address correspondence to:**

Gregory A. Poland, M.D., Director, Mayo Vaccine Research Group, Mayo Clinic, Guggenheim 611C, 200 First Street SW, Rochester, Minnesota 55905

Phone: (507) 284-4968; Fax: (507) 266-4716; Email: [poland.gregory@mayo.edu](mailto:poland.gregory@mayo.edu)

©Copyright 2017 Mayo Foundation for Medical Education and Research

**Table S1: Performance of predictive models of HAI using all three data types**.

| Input Data ^†^ | | | Feature Selection | | Continuous Prediction | | Discrete Prediction ^‡^ | | | | |
| --- | --- | --- | --- | --- | --- | --- | --- | --- | --- | --- | --- |
| Flow | mRNA | CpG | N | M | LM r^2^ | LM p-value | D ^α^ | t-test | sens | spec | AUC |
| F_0_ |  |  | 27 | 3 | 0.00 | 7.05 x 10-1 | -0.03 | 1.71 x 10-1 | 0.19 | 0.89 | 0.49 |
|  | E_0_ |  | 152 | 3 | 0.00 | 4.95 x 10-1 | 0.06 | 2.50 x 10-1 | 0.52 | 0.58 | 0.51 |
|  | F_0_^C^ |  | 15 | 6 | 0.08 | 4.50 x 10-4 | 0.49 | 1.60 x 10-1 | 0.68 | 0.65 | 0.64 |
|  | E_28-0_ |  | 31 | 3 | 0.01 | 2.42 x 10-1 | -0.08 | 4.12 x 10-2 | 0.58 | 0.53 | 0.55 |
|  | E_28-0_ ∩ F_0_^C^ |  | 63 | 0 |  |  |  |  |  |  |  |
|  |  | M_0_ | 488 | 3 | 0.00 | 5.93 x 10-1 | 0.18 | 6.08 x 10-1 | 0.78 | 0.33 | 0.54 |
| F_0_ | E_0_ |  | 179 | 2 | 0.01 | 2.11 x 10-1 | -0.01 | 4.65 x 10-1 | 0.48 | 0.62 | 0.53 |
| F_0_ | F_0_^C^ |  | 42 | 3 | 0.00 | 8.00 x 10-1 | -0.27 | 8.50 x 10-1 | 0.71 | 0.44 | 0.54 |
| F_0_ | E_28-0_ |  | 58 | 2 | 0.00 | 3.94 x 10-1 | -0.12 | 9.88 x 10-2 | 0.74 | 0.44 | 0.56 |
| F_0_ | E_28-0_ ∩ F_0_^C^ |  | 29 | 2 | 0.01 | 2.32 x 10-1 | -0.56 | 2.40 x 10-1 | 0.58 | 0.55 | 0.57 |
| F_0_ |  | M_0_ | 515 | 2 | 0.03 | 3.59 x 10-2 | 0.20 | 9.52 x 10-1 | 0.46 | 0.73 | 0.58 |
| F_0_ | E_0_ | M_0_ | 667 | 2 | 0.01 | 2.53 x 10-1 | -0.01 | 6.95 x 10-1 | 0.77 | 0.41 | 0.56 |
| F_0_ | F_0_^C^ | M_0_ | 530 | 2 | 0.00 | 5.91 x 10-1 | 0.02 | 7.39 x 10-1 | 0.47 | 0.67 | 0.52 |
| F_0_ | E_28-0_ | M_0_ | 546 | 2 | 0.00 | 8.28 x 10-1 | 0.04 | 4.20 x 10-1 | 0.62 | 0.53 | 0.55 |
| F_0_ | E_28-0_ ∩ F_0_^C^ | M_0_ | 517 | 3 | 0.02 | 6.04 x 10-2 | -0.10 | 4.46 x 10-1 | 0.81 | 0.55 | 0.66 |
| Best Representative ^β^ | | |  |  |  |  |  |  |  |  |  |
|  | E_0_, k=25 | M_0_, k=25 | 50 | 4 | 0.02 | 6.45 x 10-2 | 0.25 | 4.24 x 10-1 | 0.85 | 0.41 | 0.59 |
| F_0_ | E_0_, k=25 | M_0_, k=25 | 77 | 2 | 0.02 | 1.19 x 10-1 | 0.26 | 2.95 x 10-1 | 0.81 | 0.35 | 0.56 |
|  | E_0_, k=6 | M_0_, k=8 | 14 | 3 | 0.01 | 2.57 x 10-1 | 0.00 | 3.66 x 10-1 | 0.41 | 0.80 | 0.59 |
|  | E_0_, WGCNA=15 | M_0_, k=8 | 23 | 5 | 0.03 | 3.07 x 10-2 | 0.19 | 5.07 x 10-1 | 0.64 | 0.55 | 0.59 |
| F_0_ | E_0_, k=6 | M_0_, k=8 | 41 | 2 | 0.00 | 8.18 x 10-1 | -0.50 | 2.86 x 10-2 | 0.48 | 0.59 | 0.52 |
| Medoid Representative | | |  |  |  |  |  |  |  |  |  |
|  | E_0_, k=25 | M_0_, k=25 | 50 | 2 | 0.00 | 9.62 x 10-1 | 0.21 | 3.26 x 10-1 | 0.27 | 0.83 | 0.51 |
| F_0_ | E_0_, k=25 | M_0_, k=25 | 77 | 3 | 0.01 | 2.62 x 10-1 | 0.01 | 9.87 x 10-1 | 0.19 | 0.97 | 0.56 |
|  | E_0_, k=6 | M_0_, k=8 | 14 | 3 | 0.00 | 5.27 x 10-1 | 0.00 | 5.22 x 10-1 | 0.63 | 0.53 | 0.56 |
|  | E_0_, WGCNA=15 | M_0_, k=8 | 23 | 0 | 0.00 | 7.51 x 10-1 | -0.16 | 4.05 x 10-1 | 0.16 | 0.94 | 0.52 |
| F_0_ | E_0_, k=6 | M_0_, k=8 | 41 | 3 | 0.00 | 4.72 x 10-1 | -0.01 | 2.93 x 10-1 | 0.88 | 0.23 | 0.49 |

^†^ Abbreviations used: F_0_, day 0 Flow cell subset; E_0_, day 0 gene expression; F_0_^C^, day 0 gene expression of genes not correlated with any flow data; M_0_, day 0 methylation. N, the number of features input to the ensemble learner. M, the number of features retained in the final model. LM, linear model fit between observed and predicted outcome levels.

^‡^ For evaluative purposes, outcome data was discretized by above or below the median.

^α^ Cohen’s D statistic measuring the standardized difference in means.

^β^ Data was clustered into k clusters prior to model construction and one representative from each was used.


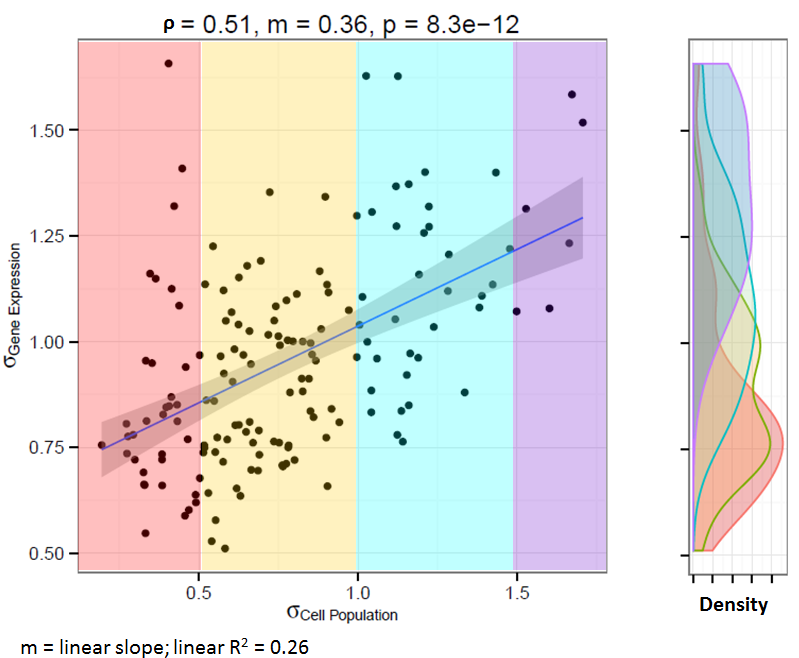


**Figure S1: PBMC-derived cell subset variability has a strong global relationship to variability in gene expression**. We computed the standard deviation of differential flow subset levels (Day 28 – Day 0) and of differential gene expression, per individual. These two metrics are plotted against one another and each point indicates the relationship between an individual’s variability in PBMC composition (assayed by flow) and differential gene expression. Spearman’s correlation coefficient between these two measures of variability is 0.51 and the linear fit is significant (p = 8.3x10^-12^). To further emphasize the impact of variability in flow levels on variability in gene expression, we split the dataset into four groups, indicated by color, and plotted the probability density of subjects in the right-hand panel.


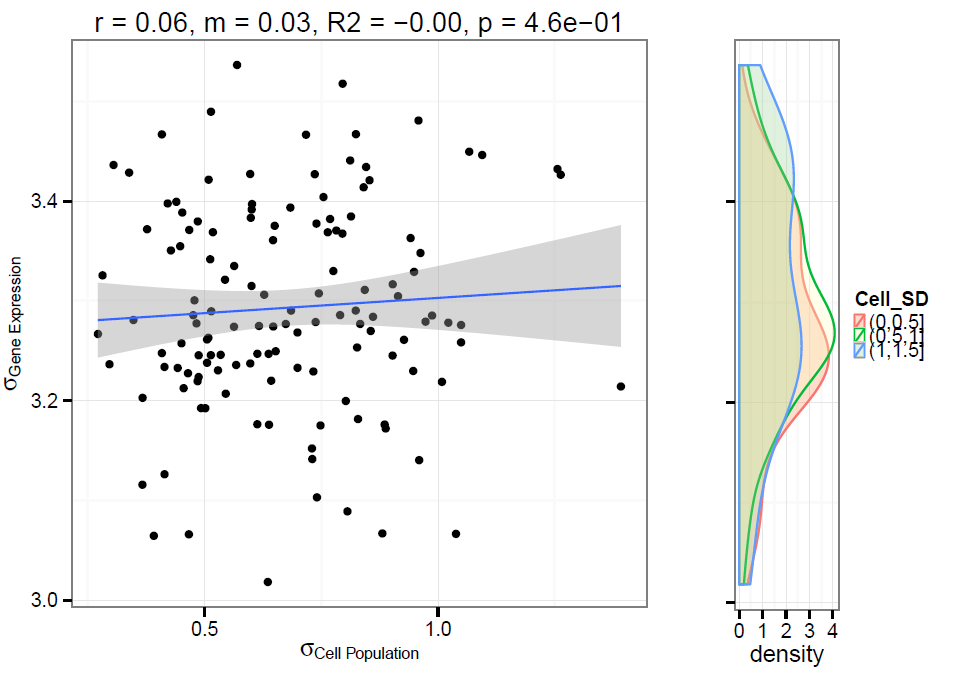


σ (Methylation M-Value)

**Figure S2**: **PBMC-derived cell subset variability has no global relationship with M-value variability.** Presentation is similar to Figure S1 and the analogous procedure was performed. No significant statistical relationship is identified between variability in flow-derived PBMC composition and variability in methylation M-levels (p = 0.46).


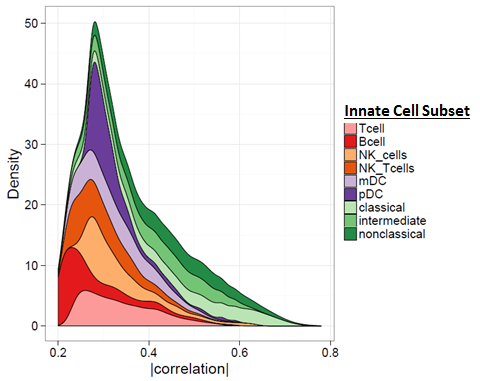


**Figure S3: Probability densities of gene expression correlating with each cell subset.** A stacked representation is used such that the total shape represents the probability density function across all genes, but the striations within correspond to cell subsets.


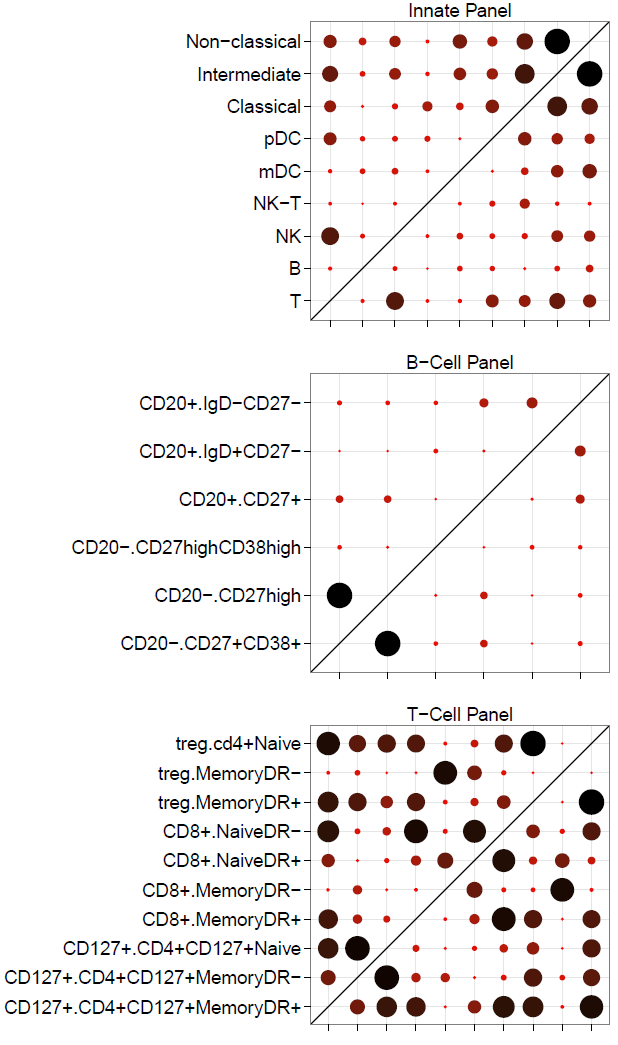


**Figure S4: Comparison of the overlapping genes correlating with each cell subset**. For each of our three flow panels, we consider the 5% (n=710) of genes most highly correlated with the change in each cell subset’s level. We show the pairwise concordance of these lists of genes, where both the color and circle diameter scale with the fraction of genes shared between any two pairs; minimal overlap is denoted by a red dot and complete overlap would be a black unit circle.


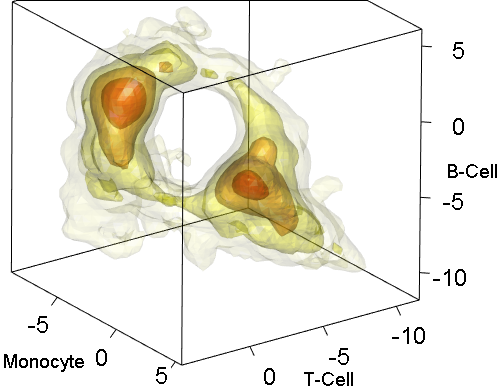


**Figure S5: Differential expression across sorted cell subsets emphasizes inter-cell-type variability.** Expression levels from our filtered cell subsets are shown using a 3D plot where each axis is a filtered cell subset and volumetric surfaces summarize the number of genes within each region. Surfaces are drawn around 95% (light yellow), 75% (yellow), 50% (orange), and 25% (dark orange) of genes. Two predominant groups of genes are evident: those that are high in monocytes but low in B- and T-cells (lower right), and those that exhibit the opposite pattern.


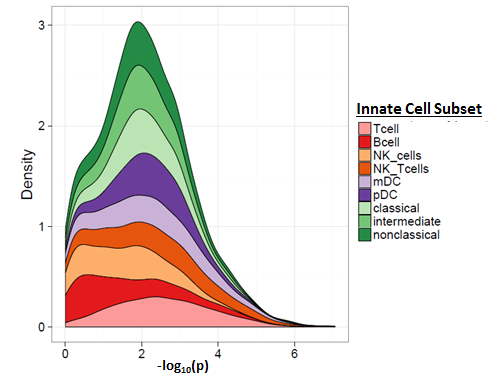


**Figure S6: Probability of flow subset-associated genes to also associate with B-cell ELISPOT outcome levels.** A similar representation to Figure S3 is used, but the abscissa is the p-value from Spearman’s correlation with B-cell ELISPOT responses, limited to genes associating with each flow-derived subset. Many of the flow subset-associated genes are also associated with B-cell ELISPOT at the p < 1x10^-2^ level, but the relative fraction of genes is different for each subset.


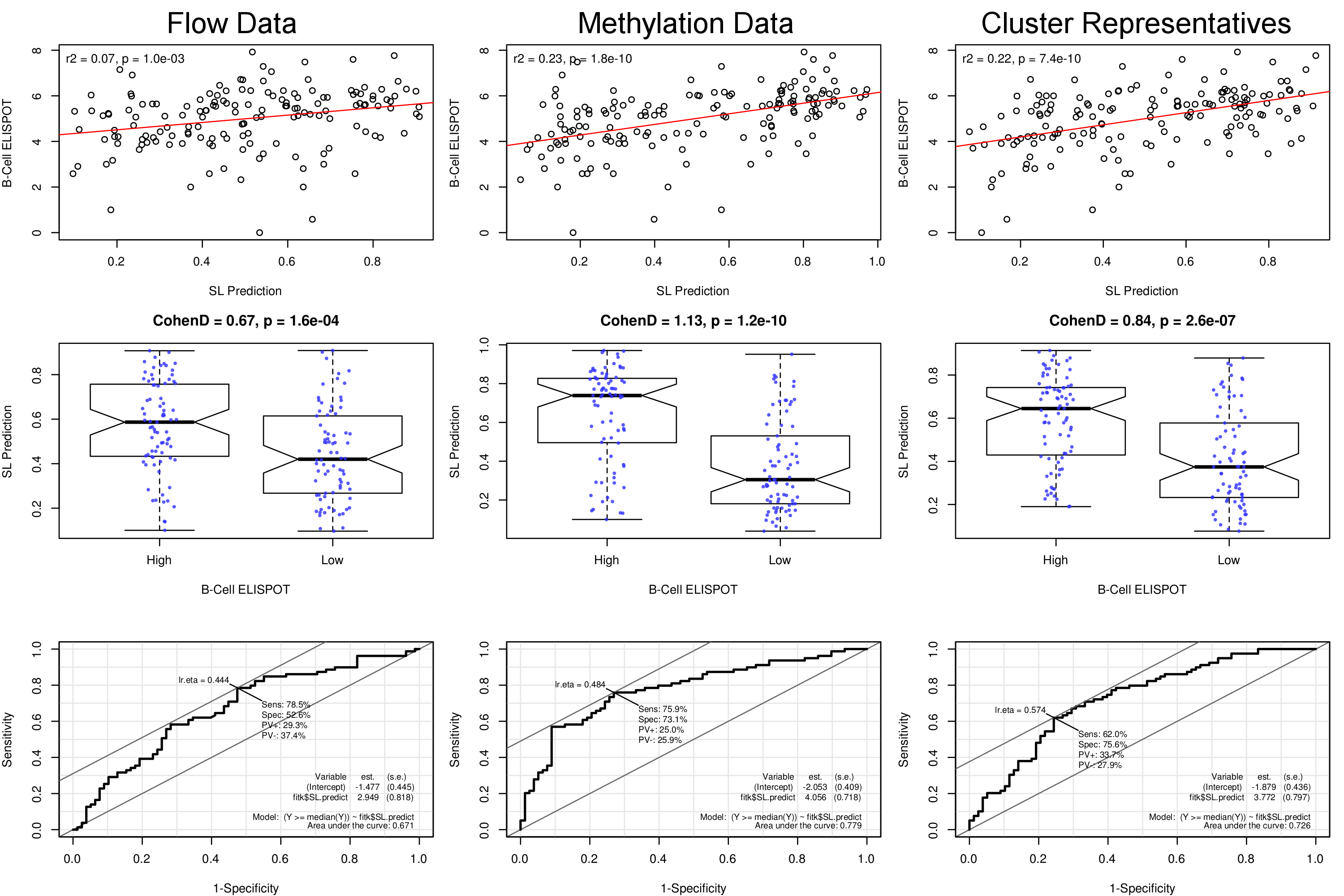


**Figure S7: Performance of selected models for predicting B-cell ELISPOT response levels.** Models selected used A) only flow data, B) only filtered CpG methylation data, or C) cluster representatives from k-means clustering (k=25) of both mRNA and CpG methylation data. The top panels show the relationships between our predictions on the abscissa and the experimentally measured values on the ordinate. The middle panels show the distribution of predicted values for samples discretized into high or low response groups based on their experimentally measured values. The lower panels show performance curves for our predictive models.


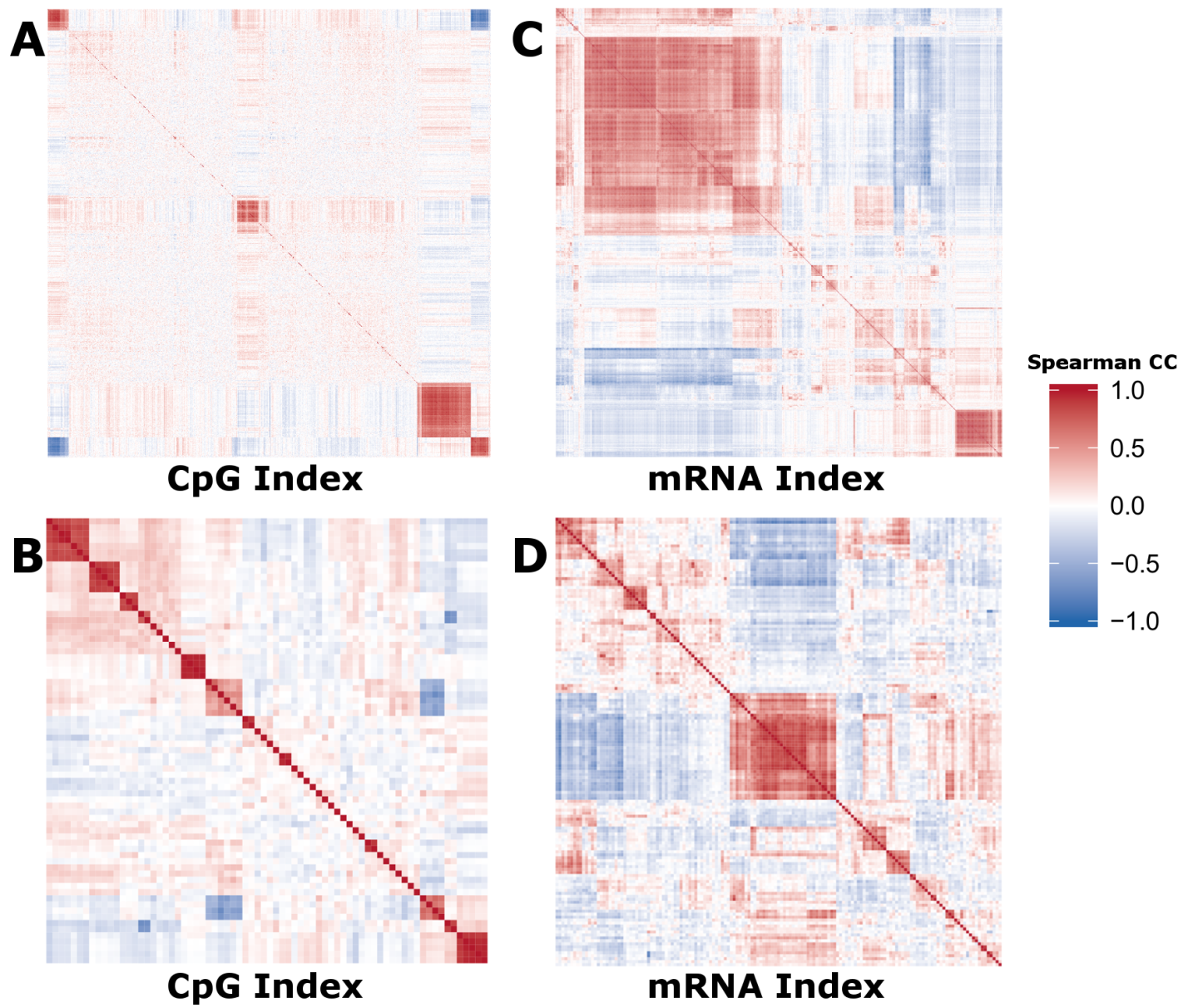


**Figure S8: Strong interdependencies are present within gene expression and methylation datasets**. We show the Spearman’s correlation coefficient among (A, B) CpG methylation sites and (C, D) gene expression levels. Correlation matrices are plotted as heat maps after our initial variance filter (A,C) and after our final variance filter (B,D).

**
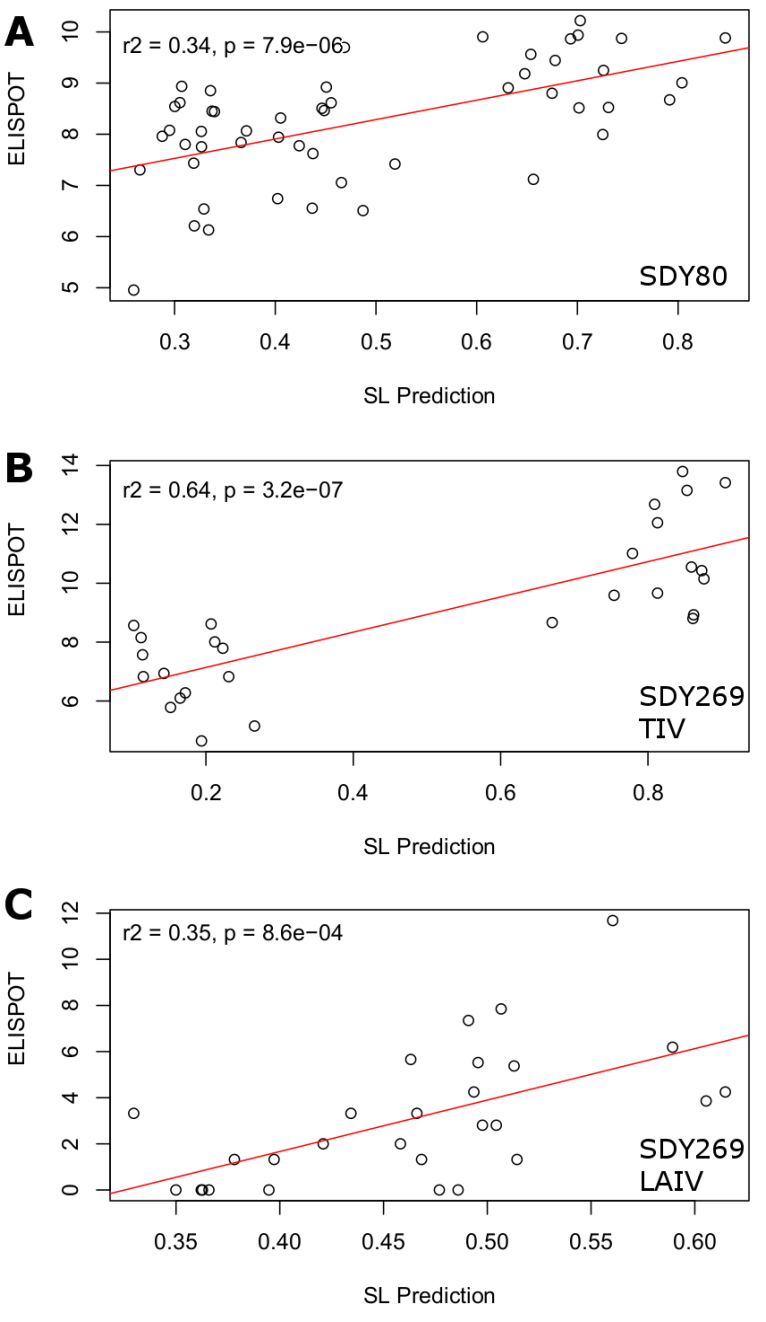
Figure S9: Genes recurrently used in predictive models of B-cell ELISPOT levels in our study predict influenza-specific ELISPOT levels in previous vaccination studies.** Models were generated using the genes recurrently identified in our predictive models, for each of the previous studies. New models were generated as our study used RNA-Seq and identified genes that were not represented on the previously used microarrays. In all three previous studies, predictive models could be generated using only these genes: *BHLHE41*, *NXPH4*, *PKIB*, *TKTL1*, *IFI44L*, *CD34*, *HSD17B14*, *MACROD2*, and *COBL*. **A-C)** Linear model fits are shown as red lines. The corresponding r^2^ and p-value are also shown on the plot. Cohen’s D statistics for our SuperLearner (SL) predictions of high versus low (median threshold) ELISPOT response levels are, respectively, 1.5, 1.4, and 1.2.

**
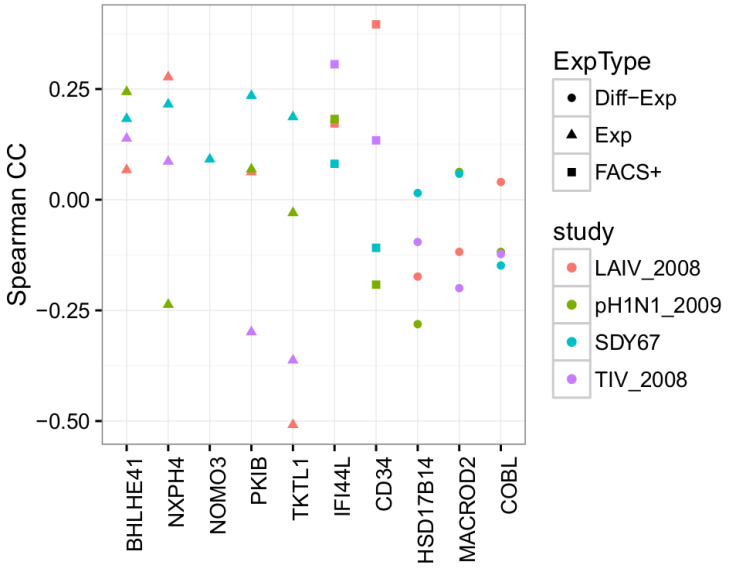
D)** The correlation between each gene’s expression level and ELISPOT outcome is plotted.

**D**
